# Using SAD data in *Phaser*

**DOI:** 10.1107/S0907444910051371

**Published:** 2011-03-05

**Authors:** Randy J. Read, Airlie J. McCoy

**Affiliations:** a CIMR Haematology, University of Cambridge, Wellcome Trust/MRC Building, Hills Road, Cambridge CB2 0XY, England

**Keywords:** SAD phasing, likelihood, molecular replacement

## Abstract

*Phaser* is a program that implements likelihood-based methods to solve macromolecular crystal structures, currently by molecular replacement or single-wavelength anomalous diffraction (SAD). SAD phasing is based on a likelihood target derived from the joint probability distribution of observed and calculated pairs of Friedel-related structure factors. This target combines information from the total structure factor (primarily non-anomalous scattering) and the difference between the Friedel mates (anomalous scattering). Phasing starts from a substructure, which is usually but not necessarily a set of anomalous scatterers. The substructure can also be a protein model, such as one obtained by molecular replacement. Additional atoms are found using a log-likelihood gradient map, which shows the sites where the addition of scattering from a particular atom type would improve the likelihood score. An automated completion algorithm adds new sites, choosing optionally among different atom types, adds anisotropic *B*-factor parameters if appropriate and deletes atoms that refine to low occupancy. Log-likelihood gradient maps can also identify which atoms in a refined protein structure are anomalous scatterers, such as metal or halide ions. These maps are more sensitive than conventional model-phased anomalous difference Fouriers and the iterative completion algorithm is able to find a significantly larger number of convincing sites.

## Abstract 2

SAD data can be used in *Phaser* to solve novel structures, supplement molecular-replacement phase information or identify anomalous scatterers from a final refined model.

## 1. Introduction

In the early days of protein crystallography, when only weak sealed-tube X-ray sources were available and diffraction intensities were measured (sometimes by eye) from photographic film, phase information could only be determined reliably if there were large intensity differences, such as from isomorphous replacement with heavy metals. To resolve the phase ambiguity inherent in phases determined from only two intensities, it was necessary to collect data from several derivatives (hence multiple isomorphous replacement; MIR). As X-ray sources and detectors have improved, allowing the intensity data to be measured much more precisely, smaller signals such as those from anomalous diffraction have become sufficient. The introduction of density-modification methods, such as solvent flattening (Wang, 1985 ▶), made it possible to resolve the phase ambiguity without adding information from multiple wavelengths or multiple heavy-atom derivatives.

These trends have led to a renaissance in single-wavelength anomalous diffraction (SAD) experiments (Dauter *et al.*, 2002 ▶), which had initially been used only rarely after the landmark demonstration of sulfur-SAD phasing for crambin (Hendrickson & Teeter, 1981 ▶). At present, nearly half of the structures determined by experimental phasing methods are solved using just SAD data. Because the success of SAD phasing depends on extracting a relatively small signal reliably and robustly, it is important to account properly for the sources of error in the experiment and to make optimal use of the data. To achieve this goal, we apply likelihood-based methods to SAD phasing.

## 2. Understanding the SAD likelihood target

**Figure 1:**
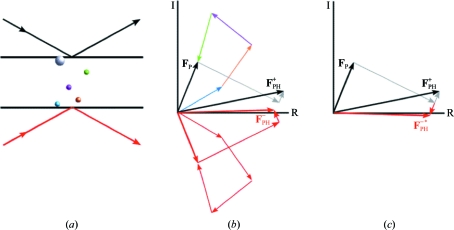
Physics of the SAD experiment. (*a*) Four normal atoms and one anomalous scatterer are shown relative to a pair of Bragg planes. Incident and diffracted X-rays for measurement of diffraction from the top of the Bragg planes (the ‘plus’ hand of a pair of measurements) are shown as black arrows, while red arrows show incident and diffracted X-rays for measurement of diffraction from the same Bragg planes but from the bottom of the planes (the ‘minus’ hand). (*b*) For the ‘plus’ hand, the phase of the contribution from the normal scatterers varies from 0 for atoms on the bottom plane to 2π for atoms on the top plane. Arrows representing their contributions to diffraction are shown by arrows in colours matching the atoms in (*a*). The anomalous scatterer has a large normal component, but because of the phase lag there is a small component perpendicular to the normal component, rotated in the counterclockwise direction. For the ‘minus’ hand, the phase of the contribution from normal scatterers has the opposite sign, varying from 0 for the top plane to 2π for the bottom plane, so their contributions (shown with red arrows) are mirrored across the horizontal axis. The normal contribution from the anomalous scatterer is also mirrored, but the phase lag again leads to a perpendicular component rotated counterclockwise, thus breaking the mirror symmetry. (*c*) The contributions for the ‘minus’ hand are reflected across the horizontal axis (giving the complex conjugate of the structure factor), showing more clearly how the anomalous scattering component of the anomalous scatterer breaks the symmetry, leading to different intensities depending on whether diffraction is measured from above or below the Bragg planes.

At typical wavelengths, most atoms (*e.g.* the C, N and O atoms of proteins) are far from an absorption edge, so that diffraction from these atoms shares a common phase shift. Atoms near an absorption edge are referred to as anomalous scatterers because their contribution to the diffraction pattern has a significant relative phase lag. In fact, all atoms have some anomalous scattering at all wavelengths, but when the anomalous scattering contribution is very small it can safely be ignored. For convenience, we refer to atoms lacking significant anomalous scattering as ‘normal’ atoms. Fig. 1 ▶ outlines the physics of the SAD experiment, showing that if a crystal contains a mixture of normal atoms and anomalous scatterers then the amplitude of diffraction observed from a set of Bragg planes differs depending on whether the incident and diffracted X-rays are on one side of the crystal or the other, *i.e.* corresponding to the plus and minus hands of the Miller indices describing the Bragg planes.

**Figure 2:**
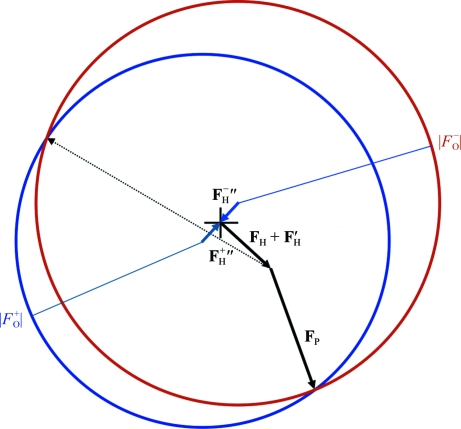
The conventional Harker construction for SAD phasing. The total structure factors for **F** ^+^ and **F** ^−^* (the complex conjugate of **F** ^−^) are sums of complex numbers (which can be represented as vectors) with common components. In the Harker construction we represent **F** ^+^ as the vector sum of the imaginary contribution from the anomalous scatterers (**F**_H_^+^′′), the real contribution from the anomalous scatterers (**F**_H_ + **F**_H_′) and the unknown contribution from the rest of the protein (**F**_P_, represented with two possibilities in solid and dashed arrows). Since the amplitude of the total structure factor, |*F*_o_^+^|, is known, the **F**_P_ vector must end up on the blue circle, which is centred on the tail of the **F**_H_^+^′′ vector and has a radius of |*F*_o_^+^|. Similarly, **F** ^−^* is represented as a vector sum, starting with its imaginary contribution from the anomalous scatterers (**F**_H_^−^′′) and then sharing the remaining real scattering components. The red circle, which is centred on the tail of the **F**_H_^−^′′ vector and with a radius of |*F*_o_^+^|, crosses the blue circle at the two possible values for **F**_P_; the shorter of the two possible vectors is more probable. If the structure factor will be used for a map containing the anomalous scatterers, the origin of the Harker construction is taken at the base of the vector for the real contribution from the anomalous scatterers, indicated by a cross.

Fig. 2 ▶ provides a Harker construction that illustrates two important features of SAD phasing. Firstly, if the circles intersect then the experimental data are essentially compatible with the model of the anomalous scatterers, from which the offset of the two circles can be calculated. Secondly, the two points of intersection define the two phase angles that are most consistent with the experimental data and the anomalous scatterer model. In addition, it can be seen from this figure that if the structure-factor contribution from the anomalous scatterer model is closer to one of the two points where the circles intersect, the phase corresponding to that closer point of intersection will be more probable. This is because the structure factor for the remaining protein component (which makes up the vector difference between the anomalous scatterer contribution and the intersection point) has a Wilson probability distribution (Wilson, 1949 ▶), for which smaller structure factors are more probable.

**Figure 3:**
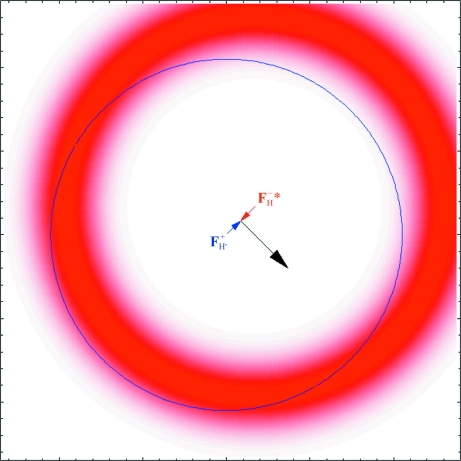
The probabilistic Harker construction for SAD phasing. For this figure, the base of the **F**_H_^+^′′ vector is chosen as the origin. Uncertainty in the anomalous scatterer model will lead to uncertainty in the scale and orientation of the set of black, red and blue vectors representing the real and imaginary contributions of the anomalous scatterers to **F** ^+^ and **F** ^−^*. This leads to uncertainty in the position of the red circle, which is represented as a circular distribution of red shading. The contribution of errors in the measurement of the observed |*F*_o_^+^| and |*F*_o_^−^| can be represented as a further increase in the width of the red distribution.

The conventional Harker construction makes no allowance for experimental errors in the measured amplitudes or for errors in the model of the anomalous scatterers. Measurement errors lead to uncertainty in the radii of the circles, which can be represented by smearing out the circles; there are no longer two defined crossing points but rather a range of phase angles corresponding to different levels of overlap between the circular distributions. Conveniently, it turns out to be mathematically equivalent in computing a likelihood target to combine the error from both measurements and smear out only one of the circles. Errors in the anomalous scatterer model lead to uncertainty in the structure factor computed from the model and hence to uncertainty in the offset between the circles. If we use one of the pair of structure factors as our reference point, then the model uncertainty leads to further smearing of the circle corresponding to the second structure factor. The probabilistic Harker construction is illustrated in Fig. 3 ▶ and an animation illustrating the effect of model errors is provided in the supplementary material.^1^

The SAD likelihood function (McCoy *et al.*, 2004 ▶) is the joint distribution of the amplitudes for the plus and minus hands given the contributions computed from the anomalous scatterer model. This is computed from the joint distribution of the two (complex) structure factors by integrating over their possible phases. The joint structure-factor distribution, in turn, can be factored into a product between the probability of one of the two structure factors given the corresponding contribution computed from the anomalous scatterer model and the probability of the second structure factor given the first and both calculated structure factors.

These two components can be identified with the considerations discussed above. The probability of one structure factor given the contribution from the model will usually be dominated by the Wilson distribution of the protein contribution to the structure factors, which can partially resolve the ambiguity between the two most probable phases described by the distribution of the second structure factor given the first.

## 3. Experimental phasing with SAD

### 3.1. Initial data analysis

Data are corrected for anisotropy (McCoy *et al.*, 2007 ▶) and then placed approximately on an absolute scale using an algorithm similar to that used in the program *BEST* (Popov & Bourenkov, 2003 ▶). Because the presence of outliers can distort the likelihood target, two outlier tests are applied. Firstly, the *F* ^+^ and *F* ^−^ measurements are both checked for implausibly large values using a test based on the Wilson distribution (Read, 1999 ▶). Secondly, the size of the anomalous difference is checked by computing the probability of one of the pair of measurements given the other. If any of these probabilities is too low (with a threshold set by default at one in a million), the pair of observations is rejected for that cycle of refinement and phasing. However, as the estimates of the variances in the SAD likelihood target are refined the outlier tests are repeated periodically.

### 3.2. Refinement and phasing

Phasing in *Phaser* starts from an initial substructure, which can be obtained by using one of the dual-space methods such as *SnB* (Miller *et al.*, 1994 ▶), *SHELXD* (Sheldrick, 2008 ▶) or *HySS* (Grosse-Kunstleve & Adams, 2003 ▶). The SAD likelihood target is optimized by refining, by default, the positions, occupancies and atomic displacement parameters of the atoms in the model, as well as variances describing the errors arising from missing scattering in the real scattering model and errors in the prediction of one member of the Friedel pair from the other. If the wavelength is close to an absorption edge then by default the *f*′′ for that anomalous scatterer is refined as well. Optionally, users can choose to refine a scale factor applied to the estimated standard deviations of the amplitude measurements.

To stabilize the simultaneous refinement of occupancies, *B* factors and *f*′′, the isotropic part of the *B* factor is restrained to be similar to the overall Wilson *B* factor for the data set (obtained from the calculations to place data on an absolute scale) and *f*′′ is restrained to be similar to its initial value, obtained either by table lookup from the wavelength (Sasaki, 1989 ▶) or by user input. Note, however, that *f*′′ is only refined by default if the wavelength is near the absorption edge of the element. A sphericity restraint adds a penalty that prevents anisotropic *B* factors from becoming highly anisotropic unless it is required to explain the diffraction data.

Phase probabilities for acentric reflections with Friedel pairs are computed from the integrand of the SAD likelihood target (McCoy *et al.*, 2004 ▶). For singletons (acentric reflections for which only one of *F* ^+^ or *F* ^−^ has been measured) and centric reflections, phase information comes essentially from the real scattering of the anomalous scatterer model and is computed in the same way as phase probabilities for any partial models (Read, 1986 ▶).

### 3.3. Log-likelihood gradient substructure completion

When the initial substructure is determined, the dual-space methods usually use the anomalous differences as approximate estimates of the structure-factor contribution from the anomalous scatterers and there is no mechanism to account for the relative measurement errors of different observations. In addition, the user must make an initial guess of the number of anomalous scatterers to be expected. This can be an overestimate if the residues containing intrinsic anomalous scatterers (Se in Met or S in Met or Cys) are disordered or an underestimate if there is static disorder of these residues; for halide soaks only a rough guess of the number of sites can be made.

**Figure 4:**
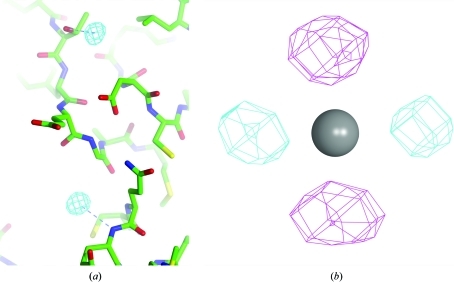
Log-likelihood gradient (LLG) maps, contoured at +6 (cyan contours) and −6 (magenta contours) times the r.m.s. deviation of the LLG map; this figure was prepared using *CCP*4*mg* (Potterton *et al.*, 2004 ▶). (*a*) Using data from a bromide soak of the human acyl protein thioesterase I (Devedjiev *et al.*, 2000 ▶), the program *HySS* (Grosse-Kunstleve & Adams, 2003 ▶) found a substructure of 21 bromide ions. After refinement in *Phaser* (in which the occupancies refined close to zero for six of the sites), an LLG map was computed. Density is shown for the top two sites in the context of the final protein model, which was not consulted in the calculation. At the convergence of LLG completion, the substructure contained 40 sites. (*b*) A model of the four Yb atoms in the Yb-substituted mannose-binding protein (Burling *et al.*, 1996 ▶) was refined in *Phaser* with isotropic *B* factors before computing an LLG map, which illustrates the positive and negative features that indicate anisotropic motion.

In *Phaser*, the substructure is com­pleted by using log-likelihood gradient (LLG) maps (McCoy & Read, 2010 ▶) similar in concept to those used for isomorphous derivatives in the program *SHARP* (de La Fortelle & Bricogne, 1997 ▶). Because the underlying SAD likelihood target accounts for the measurement errors in the individual observations, the LLG maps are robust to experimental error. No assumptions need to be made about the number of sites: sites are deleted if the atoms refine to low occupancies or added when there is a sufficiently large peak in the LLG map that is not too close to an existing atom. By default, peaks above six times the r.m.s. deviation of the LLG map, *i.e.* with a *Z* score above 6, are considered significant. As iterative substructure completion proceeds, the errors become smaller and as a result the LLG maps become more sensitive to minor sites. LLG maps are also used to detect anisotropy. If a significant peak or hole is found near an existing atom then that atom is flagged for anisotropic refinement. Fig. 4 ▶ shows examples of LLG maps indicating new or anisotropic sites.

**Figure 5:**
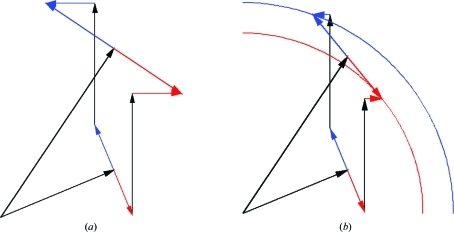
Breakdown of Friedel’s law applied to scattering contribution of mixed anomalous substructure. (*a*) shows that Friedel’s law is obeyed for the plus and minus hands of partial structure factors obtained by adding the contributions of two atoms that have the same ratio of real to imaginary scattering. In contrast, (*b*) shows that Friedel’s law breaks down for the plus and minus hands of partial structure factors obtained by adding the contributions of atoms that differ in their ratio of real to imaginary scattering.

Because the LLG maps can be computed for more than one type of anomalous scatterer, taking into account the relative size of the real (normal) and imaginary (anomalous) scattering, LLG completion can define a substructure comprising a mixture of atom types. If a peak is found in more than one LLG map, the atom type is initially identified by which map gave the highest *Z* score. This preliminary identification can be revised if the occupancy refines to physically unrealistic values (McCoy & Read, 2010 ▶). Note that when the substructure contains more than one type of anomalous scatterer the refined likelihood can indicate which hand is correct for the substructure. This can be understood by reference to Fig. 5 ▶, which shows that Friedel’s law is obeyed for the scattering contribution of a substructure composed purely of one type of anomalous scatterer but is broken when the substructure contains a mixture of different types of scatterer.

## 4. Using a partial model to find anomalous scatterers

Although we have been referring to an atomic model of the anomalous substructure, there is nothing in the derivation of the SAD likelihood target demanding that the atoms in the model have a significant anomalous component to their scattering. The likelihood target applies equally well when the atoms in the model are all real scatterers.

This opens a new application for the SAD likelihood target in *Phaser*. A protein model composed of real scatterers can be used as the initial model and LLG maps can then be used to define the substructure of anomalous scatterers. (In fact, if the data extend to atomic resolution *Phaser* is capable of completing the structure with real scatterers.) There are three different scenarios where it is useful to start from a partial protein model.

### 4.1. SAD phasing from a molecular-replacement solution

If the anomalous signal is relatively weak, the dual-space substructure-determination methods can fail to find the correct substructure. Nonetheless, the anomalous signal may still provide useful phase information if the substructure can be defined. If even a poor molecular-replacement model is available, LLG completion from the molecular-replacement model can succeed in determining the substructure. Because the molecular-replacement model is just part of the total model used in maximizing the SAD likelihood target, the resulting phases automatically combine the information from the molecular-replacement model with the information from the anomalous differences, with correct relative weights.

The potential benefits of this strategy can be seen using data that we have made available for use in tutorials (http://www.phaser.cimr.cam.ac.uk/index.php/Tutorials). A set of data were collected on hen egg-white lysozyme using our home X-­ray source, but the cryocooling failed before high redundancy was obtained. We have been unable to determine the substructure from these data using tools such as *SHELXD* and *HySS*. The structure can be solved by molecular replacement using goat α-lactalbumin (PDB code 1fkq; Horii *et al.*, 2001 ▶), a relatively poor model that shares 45% sequence identity. When the molecular-replacement model is used to initiate LLG substructure completion, *Phaser* finds all ten S atoms plus several bound chloride ions. The resulting map is significantly easier to interpret than the map obtained using only molecular-replacement phases.

### 4.2. Iterating substructure determination from a preliminary atomic model

In some cases, the substructure determined by dual-space methods followed by LLG completion is still incomplete, leading to suboptimal phasing. If the map is sufficient to build a partial preliminary model using a program such as *ARP*/*wARP* (Langer *et al.*, 2008 ▶), *PHENIX AutoBuild* (Terwilliger *et al.*, 2008 ▶) or *Buccaneer* (Cowtan, 2006 ▶), then this model can be used to re-initiate substructure determination.

One clear example is given by the structure of *Escherichia coli* nitrate reductase A (Bertero *et al.*, 2003 ▶), which was solved by a combination of Fe-MAD and MIRAS phasing. It is possible to solve this structure by SAD phasing from the Fe absorption peak data alone, particularly if an iterative phasing strategy is used (as described in detail in McCoy & Read, 2010 ▶). The enzyme contains a number of 4Fe–4S clusters, haem groups and an Mo atom, as well as a large number of S and P atoms that have significant anomalous signal at the Fe peak wavelength of 1.7325 Å. After using *HySS* to find Fe atoms and completing this preliminary substructure with Fe, Mo and S atoms, the substructure used for initial phasing contains 57 atoms. The map is sufficient to trace about 70% of the structure (approximately 2000 residues) with automated building in *ARP*/*wARP* (Langer *et al.*, 2008 ▶), but when sub­structure determination with *Phaser* is iterated from this partial model the number of anomalous scatterers increases to 105 and a second round of model building traces over 90% of the structure.

### 4.3. Identifying anomalous scatterers in the final refined model

Weiss and coworkers have demonstrated that with careful data collection it is possible to verify the positions of intrinsic anomalous scatterers at the end of refinement using model-phased anomalous difference Fourier maps (Mueller-Dieckmann *et al.*, 2007 ▶). Some of these sites, including bound halides, may otherwise be difficult to identify. SAD LLG maps, starting from the refined model, should be even more sensitive in detecting the positions of anomalous scatterers for two reasons. Firstly, the SAD target takes proper account of experimental errors, which are ignored in the anomalous difference Fourier. Secondly, LLG completion is an iterative process in which including the sites that are identified in early rounds should improve the signal for identifying weaker sites in subsequent rounds. In collaboration with Manfred Weiss and Christoph Mueller-Dieckmann, we are looking at the 23 data sets that they described earlier (Mueller-Dieckmann *et al.*, 2007 ▶).

A clear indication of the potential of this approach is given by another test case, the complex of CK2α with a chlorinated inhibitor, DRB. Data were collected using a wavelength of 2 Å to optimize the anomalous signal for sulfur and chlorine. An anomalous difference Fourier map showed the sites of all ten S atoms, the four Cl atoms from two bound inhibitor molecules and an additional 18 chloride ions (Raaf *et al.*, 2008 ▶). LLG completion with *Phaser*, looking for S atoms (which are essentially indistinguishable from Cl atoms at this wavelength), finds 31 anomalous scatterer sites in the first cycle of completion; when completion converges there are 63 sites, including 20 atoms labelled as waters in the file deposited at the PDB (2rkp), two new sites and two split (partial occupancy) sites.

## 5. Practical aspects of SAD phasing with *Phaser*

### 5.1. Scattering factors

The best results are obtained when the anomalous scatterers are assigned the correct ratio of real (*f* + *f*′) to imaginary (*f*′′) scattering, because this constrains the relative contributions of the atoms to the two components of the SAD likelihood function. The wavelength for data collection must therefore be specified; if this is far from an absorption edge then the scattering factors determined by table lookup (Sasaki, 1989 ▶) will be reliable. If the wavelength is near an absorption edge, it is preferable to supply values of *f*′ and *f*′′ obtained from a fluorescence scan. By default, the initial value of *f*′′ will be refined if the wavelength is near an absorption edge, but will otherwise be fixed.

### 5.2. Asymmetric unit contents

The algorithm used to place the data on an absolute scale (similar to the algorithm described by Popov & Bourenkov, 2003 ▶) requires knowledge of the content of the asymmetric unit. This is most easily supplied through the amino-acid or nucleic acid sequences of the components of the crystal, together with the expected number of copies of each com­ponent. The plausibility of the assumed content is checked in *Phaser* against solvent-content frequencies determined from a statistical analysis of the PDB (Kantardjieff & Rupp, 2003 ▶). If the data are placed correctly on the absolute scale, then the refined occupancies of the anomalous scatterers will also be on the correct scale. It is most important to specify the correct content information when LLG completion is carried out on more than one type of anomalous scatterer, because the atom types are reassigned to give plausible occupancies. In this case, if there is any ambiguity about the number of copies of molecules in the asymmetric unit it may be worthwhile to run more than one phasing calculation, varying the assumed number of copies.

### 5.3. Using the *ccp*4*i* interface

Details of how to carry out SAD phasing using the *ccp*4*i* interface are given in the ‘Experimental phasing with *Phaser*’ section of the *CCP*4 Wiki (http://ccp4wiki.org). This also discusses the interpretation of the output, including log files and structure-factor data in the MTZ format, and provides some advice about how to use the results from *Phaser* in subsequent density-modification and model-building steps.

## Supplementary Material

Animated supplementary figure.. DOI: 10.1107/S0907444910051371/ba5159sup1.gif

Animated supplementary figure.. DOI: 10.1107/S0907444910051371/ba5159sup2.html

## Footnotes

1 Supplementary material has been deposited in the IUCr electronic archive (Reference: BA5159). Services for accessing this material are described at the back of the journal.

## References

[bb1] Bertero, M. G., Rothery, P. A., Palak, M., Hou, C., Lim, D., Blasco, F., Weiner, J. H. & Strynadka, N. C. (2003). *Nature Struct. Biol.* **10**, 681–687. 10.1038/nsb969 12910261

[bb2] Burling, F. T., Weis, W. I., Flaherty, K. M. & Brünger, A. T. (1996). *Science*, **271**, 72–77. 10.1126/science.271.5245.72 8539602

[bb3] Cowtan, K. (2006). *Acta Cryst.* D**62**, 1002–1011. 10.1107/S0907444906022116 16929101

[bb4] Dauter, Z., Dauter, M. & Dodson, E. J. (2002). *Acta Cryst.* D**58**, 494–506. 10.1107/s090744490200118x 11856836

[bb5] Devedjiev, Y., Dauter, Z., Kuznetsov, S. R., Jones, T. L. & Derewenda, Z. S. (2000). *Structure*, **8**, 1137–1146. 10.1016/s0969-2126(00)00529-3 11080636

[bb6] Grosse-Kunstleve, R. W. & Adams, P. D. (2003). *Acta Cryst.* D**59**, 1966–1973. 10.1107/s0907444903018043 14573951

[bb7] Hendrickson, W. A. & Teeter, M. M. (1981). *Nature (London)*, **290**, 107–113. 10.1038/290107a0 PMC5536114 28769131

[bb8] Horii, K., Saito, M., Yoda, T., Tsumoto, K., Matsushima, M., Kuwajima, K. & Kumagai, I. (2001). *Proteins*, **45**, 16–29. 10.1002/prot.1119 11536356

[bb9] Kantardjieff, K. A. & Rupp, B. (2003). *Protein Sci.* **12**, 1865–1871. 10.1110/ps.0350503 PMC2323984 12930986

[bb10] La Fortelle, E. de & Bricogne, G. (1997). *Methods Enzymol.* **276**, 472–494. 10.1016/S0076-6879(97)76073-7 27799110

[bb11] Langer, G., Cohen, S. X., Lamzin, V. S. & Perrakis, A. (2008). *Nature Protoc.* **3**, 1171–1179. 10.1038/nprot.2008.91 PMC2582149 18600222

[bb12] McCoy, A. J., Grosse-Kunstleve, R. W., Adams, P. D., Winn, M. D., Storoni, L. C. & Read, R. J. (2007). *J. Appl. Cryst.* **40**, 658–674. 10.1107/S0021889807021206 PMC2483472 19461840

[bb13] McCoy, A. J. & Read, R. J. (2010). *Acta Cryst.* D**66**, 458–469. 10.1107/S0907444910006335 PMC2852310 20382999

[bb14] McCoy, A. J., Storoni, L. C. & Read, R. J. (2004). *Acta Cryst.* D**60**, 1220–1228. 10.1107/S0907444904009990 15213383

[bb15] Miller, R., Gallo, S. M., Khalak, H. G. & Weeks, C. M. (1994). *J. Appl. Cryst.* **27**, 613–621.

[bb16] Mueller-Dieckmann, C., Panjikar, S., Schmidt, A., Mueller, S., Kuper, J., Geerlof, A., Wilmanns, M., Singh, R. K., Tucker, P. A. & Weiss, M. S. (2007). *Acta Cryst.* D**63**, 366–380. 10.1107/S0907444906055624 17327674

[bb17] Popov, A. N. & Bourenkov, G. P. (2003). *Acta Cryst.* D**59**, 1145–1153. 10.1107/s0907444903008163 12832757

[bb18] Potterton, L., McNicholas, S., Krissinel, E., Gruber, J., Cowtan, K., Emsley, P., Murshudov, G. N., Cohen, S., Perrakis, A. & Noble, M. (2004). *Acta Cryst.* D**60**, 2288–2294. 10.1107/S0907444904023716 15572783

[bb19] Raaf, J., Issinger, O.-G. & Niefind, K. (2008). *Mol. Cell. Biochem.* **316**, 15–23. 10.1007/s11010-008-9826-1 18607692

[bb20] Read, R. J. (1986). *Acta Cryst.* A**42**, 140–149.

[bb21] Read, R. J. (1999). *Acta Cryst.* D**55**, 1759–1764. 10.1107/s0907444999008471 10531526

[bb22] Sasaki, S. (1989). KEK Report 88-14. High Energy Accelerator Institute, Tsukuba, Japan.

[bb23] Sheldrick, G. M. (2008). *Acta Cryst.* A**64**, 112–122. 10.1107/S0108767307043930 18156677

[bb24] Terwilliger, T. C., Grosse-Kunstleve, R. W., Afonine, P. V., Moriarty, N. W., Zwart, P. H., Hung, L.-W., Read, R. J. & Adams, P. D. (2008). *Acta Cryst.* D**64**, 61–69. 10.1107/S090744490705024X PMC2394820 18094468

[bb25] Wang, B.-C. (1985). *Methods Enzymol.* **115**, 90–112. 10.1016/0076-6879(85)15009-3 4079800

[bb26] Wilson, A. J. C. (1949). *Acta Cryst.* **2**, 318–321.

